# ENT characteristics and therapeutic results in multisystemic disorders of mitochondrial encephalomyopathy

**DOI:** 10.1186/s40001-022-00832-7

**Published:** 2022-10-29

**Authors:** Haishan Long, Cheng Wen, Juan Zhao, Jiawei Wang, Yang Li, Xinxing Fu, Lihui Huang

**Affiliations:** 1 Department of Otolaryngology Head and Neck Surgery Key Laboratory of Otolaryngology Head and Neck Surgery (Capital Medical University) Ministry of Education, Beijing Tongren Hospital, Capital Medical University, Beijing, 100730 China; 2 Beijing Institute of Otolaryngology, Beijing, 100005 China; 3 Departments of Neurology, Central Laboratory, Beijing Tongren Hospital, Capital Medical University, Beijing, 100730 China; 4 Department of Ophthalmology Beijing Institute of Ophthalmology, Key Laboratory of Ophthalmology and Visual Science (Capital Medical University) Ministry of Education, Beijing Tongren Eye Center, Beijing Tongren Hospital, Capital Medical University, Beijing, 100730 China

**Keywords:** Hearing loss, Dysphagia, Facial weakness, Ptosis, Exercise intolerance, Mitochondrial

## Abstract

Here we report the evaluation of the frequency of subjective and objective otolaryngologic findings and therapeutic results in 32 patients with mitochondrial encephalomyopathy (MEM) from September 2001 to June 2021. Our analysis included studying the patients’ family histories, the clinical manifestations of MEM, and the therapeutic effects of treatments. The patients’ ages ranged from 2 to 77 years, with a median age of 12.3 years. We found that MEM ENT symptoms were characterized by hearing loss, dysphagia, and facial weakness. Most cases of sensorineural hearing loss were bilateral symmetrical progressive or sudden deafness since adolescence, which were often underestimated. Associated neuromuscular symptoms required mtDNA testing. Dysphagia and facial weakness occurred preferentially in middle-aged patients, and muscle biopsies were advised. Distortion product otoacoustic emissions and auditory brainstem responsetesting were more sensitive and reliable than pure tone averages for objective monitoring of pathogenesis. Administration of the mitochondrial synthase complex benefited patients with acute episodes. If patients did not fully recover and exhibitedresidual language deficits, hearing aids or cochlear implants were recommended. Counsel was given regarding synthetical treatments for facial weakness, endoscopic circopharyngealmyotomy for dysphagia, and surgical correction of ptosis. This study demonstrates that increased awareness of these symptoms is important to address appropriate interventions and avoid complications such as ablepsia, aphasia, social isolation, malnutrition, aspiration pneumonia, and heart failure in the setting of MEM.

## Introduction

Numerous studies have investigated how mitochondrial disorders (MIDs) affect tissues that generate the highest oxygen demand and have the highest energy requirement [1]. MIDs are associated with encephalopathy, audio-optic neuropathy, skeletal myopathy, cardiomyopathy, and gastrointestinal myopathy, either occurring alone or overlapping [2]. Mitochondrial encephalomyopathy(MEM) is a clinically heterogeneous group of disorders that arise from oxidative phosphorylation dysfunction of the mitochondrial respiratory chain related to mtDNA or nuclear DNA defects. A study reported that the adult mtDNA mutation rate is 1/5000, and the mutation rate of nuclear genes is 2.9/100,000 [3]. Although there are no epidemiological data on mitochondrial disease, mtDNA mutations are the most common cause of hereditary optic neuropathy in China [4]. The leucine transfer RNA gene at position 3243 of the mtDNA L-strand appears in 1.69% of diabetic patients [5]. Up to thousands of mitochondrial diseases have been reported in multiple clinics, suggesting that the disease is not very rare [6–8]. Most patients (90.3%) diagnosed with MEM experience visual and hearing impairments. This study involving 32 MEM patients provides detailed information about the frequency of subjective and objective otolaryngologic features, ophthalmologic and neurologic manifestations, and clinical effects of MEM. We recommend a coordinated multidisciplinary team (MDT), including neurologists, ophthalmologists, otolaryngologists, and audiologists, to provide MID patients with concise diagnosis and treatments, especially for idiopathic hearing loss and slow-onset delayed deafness.

## Methods

### Patient selection

Thirty-two patients with MEM were studied from September 2001 to June 2021 in Beijing Tongren Hospital, and the clinical features of these cases were analyzed. This study was approved by the Ethics Committee of Beijing Tongren Hospital, and informed consent was obtained from all patients.

### Audiological evaluations

Puretone average (PTA) testing was performed at the Beijing Institute of Otolaryngology according to the China Society of Audiology procedures in a sound-treated booth with environmental noise of less than 20 dB(A), using the Danish Otometrics Company pure tone audiometer-Conera with TDH39 pressure ear headphones test.The results were judged according to the interpretation standard of the hearing loss level as stipulated by the WHO [9]. The air conduction threshold, bone conduction threshold, and bone air conduction difference were used to judge the type of hearing loss. A normal bone conduction threshold and an air conduction threshold of  ≥ 25 dB HL were designated as conductive hearing loss. An abnormal threshold of bone conduction and air conduction with a difference of  ≤ 10 dB HL was designated as sensorineural hearing loss(SNHL).Lastly, a bone conduction difference of  > 10 dB HL was defined as mixed hearing loss. For children under 5 years of age, the average response threshold of auditory brainstem response (ABR) and 40 Hz auditory evoked potential was used. The difference of 15 dB between the two ears occurring in at least two frequencies or the difference of 10 dB at four frequencies was defined as asymmetric hearing loss. Symmetric hearing loss was defined as a hearing difference of the same frequency  < 15 dB.

Acoustic immittance (AI) was performed in the soundproof room; the environmental noise was less than 30 dB(A), using the American Grason-Stadler Company Tympstar II middle ear analyzer, with 226 Hz as the stimulating sound. The initial pressure was + 200 da Pa, the ending pressure was −400 da Pa, and the direction was from positive to negative. The tympanogram was classified as A-, As-, Ad-, B-, and C-type according to the Jerger classification, where TypeA was normal [10]. The acoustic stapedius reflex (ASR) was performed using a listening sound of 1000 Hz, and an 85 dB sound pressure level (SPL) was given at the peak pressure point of the test ear. The acoustic reflex threshold was  > 0.03 ml and had repeatability. When the stimulus sound reached the threshold intensity, the amplitude of the acoustic reflex increased with the stimulus intensity, called the acoustic reflex. The acoustic emission threshold was normal at 70–95 dB HL.

The distortion product otoacoustic emissions (DPOAE) used the ILO96 otoacoustic emission instrument of British Otodynamics, and the operating software was ILO V6. The stimulation sequence used a click with a duration of 80 μs, and the frequency distribution from 1 to 4 kHz was relatively flat. The DNLR stimulation method was used to suppress stimulation artifacts, and the stimulation intensity was 80 dB SPL. The sampling frequency of the signal was 25.6 kHz, and the sampling time was 20 ms. DPOAE were calculated by two initial short pure tones for measuring the 2f1–f2: L1 = 65, L2 = 55 dB SPL, f2/f1 = 1.22, band-pass filtering 0.6–6 kHz, repeated 260 times. The signal strength was recorded at 2 × f1−f2 ≥ −10 dB SPL, and a signal-to-noise ratio of  ≥ 3 dB was defined as normal in the DPOAE.

ABR and cochlear micropotential (CM) was performed using the Danish International Hearing Eclipse Evoked Potential Tester with conventional parameter settings [11]. Eighty-decibel nHL was used as the initial stimulus sound intensity, and the lower 10 dB steps down to the threshold. A certain amount of 6.5% chloral hydrate solution was used according to the child’s weight, and the ABR was tested after sleeping. According to WHO standards [9], the ABR response threshold  ≤ 30 dB nHL is normal hearing, 31–40 dB nHL is mild hearing loss, 41–60 dB nHL is moderate hearing loss, 61–80 dB nHL is severe hearing loss, and  ≥ 81 dB nHL is profound hearing loss.

The speech discrimination score (SDS) was calculated in a soundproof room with environmental noise of  ≤ 45 dB(A). Mandarin speech audiometry testing materials (MST-Ms) edited by Zhang Hua at the Clinical Auditory Center of Tongren Hospital were used to test the sentence recognition rate under 70 dB SPL intensity under monosyllable and quiet conditions.

### Electroneurography (ENoG)

Facial ENoG was conducted using the Xomed Company Nerve Integrity Monitoring-2XL electromyographic evoked potentiometer with these specifications: 1 Hz; 0.2 ms stimulation wave;an intensity of 2.0–3.6 mA; each stimulation lasted 100 μs; and the stimulation frequency four times/s. The bipolar concentric needle electrode was inserted into the subcutaneous of the foramina stylomastoideum, facial nerve stem, branches, and the supraorbital fossa. Facial nerve movement means corpuscular volume was recorded for the orbicularis oculi muscle, frontal muscle, orbicularis oris muscle, and paranasal muscle. In addition, we also analyzed the CAMP wave peak amplitude and stimulation intensity, F-wave, blink reflex latency and amplitude, and facial nerve stimulation phase.

Laryngeal ENoG was performed using Danish Keypoint EMG-evoked potentials to detect the spontaneous electric potential, motor unit potential, recruitmentunit, and recurrent laryngeal nerve evoked potentialin the thyroarytenoid, posterior cricoarytenoid, and the cricothyroid muscles in different states (quiet, pronounced, and breathing).

Limb electromyography (EMG) was detected in the Department of Neurology. Electrocardiogram (ECG) was detected in the Department of Cardiology.

### Structural imaging

Temporal bone CT was performed using a Philip Brilliance 64 full-body CT scanner, first supine position spiral volume scan, scan layer thickness 0.625, matrix 512 × 512, pitch 0.625; posterior image reconstruction, suprasonorbital line was the baseline, layer thickness and spacing were both 1 mm, FOV15 cm × 15 cm, bone algorithm reconstruction, window position 700HU, and window width 4000HU.

Cranial MRI conventional sequences including non-enhanced and contrast-enhanced imaging were performed by the Adopt GE SigmaHDx3.0TMRI scanner and phased-array head coil. MRI routine line was cross-sectional rapid spin-echo T1WI, TE was 10 ms.Cross-sectional FSE T2WI, TR was 3500 ms, TE was 120 ms. Matrix was 384 × 256; it was excited twice. FOV was 18 × 18 cm; layer thickness was 5.0 mm, layer spacing was 0.5 mm. The cross-section DWI adopted the SE plane echo sequence, b value was 0.1000 s/mm^2^, TR was 6000 ms, TE was 64–76 ms, it was excited twice, FOV was 18 × 18 cm, inversion angle was 90°, the matrix was 128 × 128, layer thickness was 3.0 mm, andthe pitch was 0.3 mm. The contrast-enhancing agent was gadopentetate meglumine at a dose of 0.1 mmol/kg. It was injected through the dorsal vein of the hand using a high-pressure syringe at an injection rate of 3 ml/s. The enhanced cross-sectional, sagittal, and coronal planes were scanned using T1WI.

Muscle biopsy and mtDNA gene testing were performed by Neuropathology Labs and Neuroimmunology Labs of Peking University First Hospital.

## Results

### Demographical and clinical data

Sex and age: The patients consisted of 18 (56.2%) men and 14 (43.8%) women, men: women = 1.29:1. The onset age of MEM ranged from 2 to 77 years (median 12.3), 17 patients (53.1%) developed MEM between 4 and 30 years, and the peak onset age was 6–15 years. The interval between diagnosis and observation ranged from 6 days to 40 years. The onset age of SNHL was 2–59 years; peak onset age was 7–16 years (median 13);16 patients (84.2%) had onset before the age of 30. The onset age of facial weakness was 35–50 years (median 43), and the onset age of dysphagia was 35–59 years (median 45).

### Family history

The diagnostic criteria were divided into five MEM subtypes: chronic progressive external ophthalmoplegia (CPEO: *n* = 15,46.9%); mitochondrialencephalomyopathy, lactic acidosis, and stroke-like episodes (MELAS: *n* = 5,15.6%); Kearns-Sayre syndrome (KSS: *n* = 5,15.6%);maternally inherited diabetes and deafness (MIDD: *n* = 4,12.5%); and Leber hereditary optic neuropathy (LHON: *n* = 3,12.5%) [2–6]. These criteria were applied according to the comprehensive diagnosis of clinical manifestations and auxiliary examinations in accordance with the “Diagnosis and Treatment Guidelines for Chinese Nervous System Mitochondrial Diseases” issued by the Neurological Branch of the Chinese Medical Association in 2015 [7]. Family history revealed positive maternal genetic history for each MEM subtype: eight patients(53%) with CPEO; two patients (40%) with MELAS; two patients(40%) with KSS;four patients (100%) with MIDD; and two patients (66.7%) with LHON. Syndromes among the mothers included: visual impairment (*n* = 5), SNHL (*n* = 5), diabetes (*n* = 4), epilepsy (*n* = 3), exercise intolerance (*n* = 2), hypermicrosoma (*n* = 1), gastrointestinal MELAS (*n* = 1), andmyocarditis (*n* = 1). The children of three patients presented with syndromes (one epilepsy, one exercise intolerance, and onehypermicrosoma).

### Clinical manifestations

The multiple organ manifestations of 32 MEM patients are shown in Table 1.

**Table 1:** The multiple organ manifestations of 32 MEM patients

	CPEO *N* (%)	MELAS *N* (%)	KSS *N* (%)	MIDD *N* (%)	LHN *N* (%)	Total: *N* (%)
Onset manifestation						
Otolaryngology						
Hearing loss	6(40)	5(100)	4(80)	4(100)	0(0)	19(59.4)
Tinnitus	1(6.7)	2(40)	2(40)	1(25)	0(0)	6(18.8)
Auditory agnosia	0(0)	1(20)	0(0)	0(0)	0(0)	1(3.1)
Ophthalmology						
Ptosis	15(100)	1(20)	5(100)	0(0)	0(0)	21(65.6)
Extraocular muscle paralysis	12(80)	1(20)	5(100)	0(0)	0(0)	18(56.2)
Retinitis pigmentosa	0(0)	1(20)	4(80)	0(0)	3(100)	8(25)
Optic atrophy	0(0)	2(40)	3(60)	0(0)	3(100)	8(25)
Cataract	3(20)	3(60)	0(0)	0(0)	0(0)	6(18.8)
Eye pain	0(0)	3(60)	0(0)	0(0)	0(0)	3(9.4)
Neurology						
Exercise intolerance	12(80)	2(40)	4(80)	0(0)	0(0)	18(56.2)
Stroke-like episode	0(0)	5(100)	0(0)	0(0)	0(0)	5(15.6)
Mental retardation	0(0)	1(20)	2(40)	2(66.7)	0(0)	5(15.6)
Headache	0(0)	3(60)	0(0)	0(0)	0(0)	3(9.4)
None-Neuromuscular system						
Diabetes	0(0)	0(0)	0(0)	3(100)	0(0)	3(9.4)
Accompanied manifestation						
Otolaryngology						
Dysphagia	5(33.3)	0(0)	1(20)	0(0)	0(0)	6(18.8)
Facial weakness	4(26.7)	0(0)	0(0)	0(0)	0(0)	4(12.5)
Ophthalmology						
Hemianopsia	0(0)	3(60)	0(0)	0(0)	0(0)	3(9.4)
Glaucoma	1(6.7)	2(40)	0(0)	0(0)	0(0)	3(9.4)
Diplopia	2(13.3)	0(0)	0(0)	0(0)	0(0)	2(6.3)
Neurology						
Palpitation	3(20)	3(60)	3(60)	0(0)	0(0)	9(28.1)
Muscular atrophy	4(26.7)	1(20)	2(40)	0(0)	0(0)	7(21.9)
Myoclonic seizures	0(0)	4(80)	0(0)	0(0)	0(0)	4(12.5)
Abnormal mental behavior	0(0)	2(40)	2(40)	0(0)	0(0)	4(12.5)
Dizziness	0(0)	0(0)	0(0)	3(100)	0(0)	3(9.4)
Limb numbness	0(0)	0(0)	0(0)	3(100)	0(0)	3(9.4)
Aphasia	0(0)	2(40)	0(0)	0(0)	0(0)	2(6.3)
Insomnia	0(0)	0(0)	1(20)	0(0)	0(0)	1(3.1)
None-Neuromuscular system						
Acute fever	0(0)	3(60)	1(20)	0(0)	0(0)	4(12.5)
Hypermicrosoma	2(13.3)	1(20)	1(20)	0(0)	0(0)	4(12.5)
Low BMI	2(13.3)	1(20)	1(20)	0(0)	0(0)	4(12.5)
Vomit	0(0)	2(40)	1(20)	0(0)	0(0)	3(9.4)
Diarrhea or constipation	1(6.7)	1(20)	0(0)	0(0)	0(0)	2(6.3)
Depression	2(13.3)	0(0)	0(0)	0(0)	0(0)	2(6.3)
Kidney damage	0(0)	0(0)	0(0)	1(33.3)	0(0)	1(3.1)
Hypogonadism	1(6.7)	0(0)	0(0)	0(0)	0(0)	1(3.1)

*CPEO* chronic progressive external ophthalmoplegia, *MELAS* mitochondrialencephalomyopathy lactic acidosis and stroke-like episodes, *KSS* Kearns-Sayresyndrome, *MIDD* maternally inherited diabetes and deafness, *LHON* Leber hereditary optic neuropathy

Physical examination revealed normal bilateral auricles, external ear canals, tympanic membranes, and nasal and throat cavities. Facial weakness was noted to have asymmetry of facial movements such as frowning, moving eyebrows, smiling, blowing, chewing, whistling, and House-Brackmann gradingI-III. Dysphagia involved subjective swallowing problems with normal vocal band movements but included a prolonged cold water test level 2 [12]. Multisystem involvement is shown in Table 1.

PTA testing revealed bilateral SNHL and asymmetrical threshold curve, with 19 (59.4%) patients having hearing disturbance. The audiogram showed nine descending types, five flat types, four high-frequency hearing loss, and one low-frequency hearing loss. PTA testing revealed 13 (40.6%) normal, 5(15.6%) mild, 5(15.6%) moderate, 5(15.6%) severe, and 4(12.5%) profound (Table 2). The average hearing threshold of each subtype was as follows: CPEO, 36 dB HL; MELAS, 40 dB HL; KSS, 38 dB HL; MIDD,40 dB HL; LHON, 20 dB HL; there was no statistically significant difference in PTA between each subtype (*P* = 0.85).

**Table 2:** PTA degrees of MEM subtypes

MEM subtypes	Normal hearing ≤ 25 dB HL	PTA degrees(mean frequencies of 0.5, 1, 2,4 kHz) ^[1]^				SNHL/subtypes (*N*%)
		Mild 26–40 dB HL	Moderate 41–60 dB HL	Severe 61–80 dB HL	Profound ≥ 81 dB HL	
CPEO	7(22.6)	2(6.5)	2(6.5)	2(6.5)	2(6.5)	8/15(53.3)
MELAS	2(6.5)	0(0)	1(3.2)	1(3.2)	1(3.2)	3/5(60)
KSS	1(3.2)	1(3.2)	1(3.2)	2(6.5)	0(0)	4/5(80)
MIDD	0(0)	2(6.5)	1(3.2)	0(0)	1(3.2)	4/4(100)
LHON	3(9.7)	0(0)	0(0)	0(0)	0(0)	0/3(0)
Total	13(41.9)	5(16.1)	5(16.1)	5(16.1)	3(9.7)	19/32(59.4)

*CPEO* chronic progressive external ophthalmoplegia, *MELAS* mitochondrialencephalomyopathy lactic acidosis stroke-likeepisodes, *KSS* Kearns-Sayre syndrome, *MIDD* maternally inherited diabetes and deafness, *LHON* Leber hereditary optic neuropathy

The AI tympanogram was normal on both sides, and the ASR depended on the degree of hearing loss; normal PTA and mild hearing loss could elicit ASR in 13 (40.6%) patients; moderate to profound hearing loss could not elicit ASR in 19 (59.4%) patients. Most of the DPOAEs were abnormal and undetectable. PTA was normal, but DPOAE was abnormal in two patients. Among 13 patients with normal PTA, 10 patients with ABR could be elicited, two patients’ thresholds increased, and one patient had prolonged III wave latency. ABR I and III waves were not discerned among 19patients with abnormal PTA, including five mild and five moderate. The ABR V wave could not be elicited in five severe and three profound cases. No repeatable potential was recorded in CM. SDS and PTA were closely matched except for one MELAS patient who had a normal PTA, but SDS 80 dB SPL 0%, defined as auditory agnosia after an MRI, showed damage. The subjective and objective hearing tests for each MEM subtype are shown in Table 3.

**Table 3:** Hearing assessments of MEM subtypes

MEM subtypes	PTA *N*					PTA Ab				
	AI		DPOAE *N*	ABR *N*	SDS *N*	AI		DPOAE Ab (*N*%)	ABR Ab (*N*%)	SDS Ab (*N*%)
	T *N*	ASR *N*				T Ab	ASR Ab			
CPEO	7(21.9)	7(21.9)	6(18.8)	6(18.8)	7(21.9)	0(0)	7(21.9)	8(25)	8(25)	3(9.4)
MELAS	2(6.3)	2(6.3)	1(3.1)	1(3.1)	2(6.3)	0(0)	3(9.4)	3(9.4)	3(9.4)	2(6.3)
KSS	1(3.1)	1(3.1)	1(3.1)	1(3.1)	1(3.1)	0(0)	4(12.5)	4(12.5)	4(12.5)	2(6.3)
MIDD	0(0)	0(0)	0(0)	0(0)	0(0)	0(0)	4(12.5)	4(12.5)	4(12.5)	0(0)
LION	3(9.4)	3(9.4)	3(9.4)	3(9.4)	3(9.4)	0(0)	0(0)	0(0)	0(0)	0(0)

*N* normal, *Ab* abnormal, *PTA* pure tone average, *AI* acoustic immittance, *T* tympanogram, *ASR* acoustic stapediusreflex, *DPOAE* distortion product otoacoustic emissions, *ABR* auditory brainstem response, *SDS* speech discriminationscore

### Electromyography

There were four patients with facial ENoG abnormalities: Facial nerve movement had effects on thefrontal, orbicularis oculi, orbicularis oris, and paranasal muscles. CAMP wave peak amplitude decreased by 35–70%;F-wave was not elicited; the latency of blink reflexes was prolonged; the amplitude of the waves was reduced, and the facial nerve showed a mono-mixed phase when muscles contracted. Six patients with laryngeal ENoG were designated as abnormal: the potential sdecreased by 15% in the cricothyroid and posterior cricoarytenoid muscles, which were stimulated three times repetitively. Seven patients had abnormal limbs as verified by EMG and displayed skeletal muscle myogenic or neurogenic damage. Nine patients had abnormal ECG findings, including heart blocking, depressed ST, and flat T-wave. Other related abnormalities related to ENoG were found in patients, including the cranial video EEG showing increased 4–7c/s slow waves of invasion side; moderate rhythm disturbances; no three-phase waves; and the appearance of Q-wave short-range rhythms. Visual evoked potential (VEP) showed that the P-ERG wave on the invasion side had a prolonged P100 latency and flat waveform amplitude. The P100 latency of each wave of F-VEP was prolonged, and the amplitude of the OzO2 wave was flattened.

### Structural imaging

In our study, no abnormalities were found in CT of the temporal bone. Patients whose syndrome did not invade the central nervous system may have had expected MRI results, and various subtypes of MEM have different MRI findings. Nineteen patients with abnormal changes were found: eight patients (25.8%) with central nerve demyelination; six patients (19.4%) with white matter dysplasia; five patients (16.1%) with brain atrophy; eight patients (25.8%) with multiple lacunar infarctions; and a single case (3.2%) of basal ganglia calcification. Lesions can change their range and locations with progression in MELAS. Examination showed that the temporal lobe, hippocampus, occipital parietal lobe, and cingulate gyrus were swollen, with a corresponding enhancement of the pial meninges and cortex. MELAS was also characterized by bilateral basal ganglia, corona radiata, centrum semiovale, and multiple ischemic lesions in the white matter of the subtemporal cortex on both sides,as well as ischemic demyelination of the white matter on both sides of the ventricle [13, 14]. KSS was characterized by brain atrophy, long T2 signals in the subcortical white matter, thalamus, basal ganglia, brainstem, and multiple patchy abnormal signals around the ventricles on both sides.

### Muscle biopsy and gene test

Muscle biopsy is an important diagnostic method for subtypes of muscle damage. In our study, 16 of the 32 patients underwent biopsy, and 11 patients showed that the affected skeletal muscle had ragged red fibers, ragged blue fibers, vessels of succinate dehydrogenase(SDH), and cytochrome oxidase C stain (COX)-negative muscle fibers [15]. In the absence of fiber damage, mitochondrial myopathy could also be identified by mitochondrial-specific enzymes around the vessels, which were increased significantly. Gene testing of 32 patients was carried out in one of two generations due to the different types of mtDNA or nuclear gene mutations of various subtypes because of its unique mutation pattern. CPEO and KSS were characterized mainly by deletions of γ-polymerase genes or mtDNA fragments of nuclear DNA [15, 16]. The A3243G point mutation in mtDNA was common in MELAS and MIDD [17, 18]. LHON was mostly an mtDNA point mutation [19, 20].

### Diagnosis

According to the Guidelines [21], MEM should be considered when the following is involved: myopathy, combined with a single system involvement (may be the central nervous system); central nervous system combined with two other systems (of which one may be the muscular system); and multisystemic syndromes (at least three systems), including neuromuscular involvement. Patients with hearing impairments, such as deafness, tinnitus, and auditory agnosia, often experience other systemic impairments, such as ptosis, vision loss, muscle weakness, facial paralysis, dysphagia, intelligence retardation, and short stature. ENT specialists should consider the possibility of MEM and look for the pathogenicityina family with SNHL using evidence such as electromyography, cranial MRI, and pathogenic exams. The summaries of clinical features, audiology, electromyography, and cranial MRI can point to a clinical diagnosis of MEM, and further muscle biopsy and gene testing canhelp confirm the diagnosis, subtypes and assess the prognosis.

### Therapeutic regimens

Regarding the lesion site, SNHL patients could wear hearing aids or receive a cochlear implant when hearing aids were ineffective [22]. In our ENT clinic, three patients wore hearing aids, and SDS increased to 80 dB SPL (85.1 ± 15.1%). One KSS patient with profound SNHL experienced an SDS increase to 80 dB SPL 56% after cochlear implantation (C124RE CA. Cochlear Ltd. Nucleus C124RE device). Patients experiencing facial weakness could choose to have an autograft of fascia or muscle for static or dynamic repair. In the presence of dysphagia caused by pharyngeal muscle involvement, gastrogavage and partial pharyngeal muscle dissection could be beneficial [23]. Other comprehensive treatments included ahigh-energy diet [24], aerobic endurance exercise [25] (except for those with fever, muscle pain, or in a hunger state), and physiotherapy [26]. Patients who underwent surgery should have intravenous sugar-containing fluids administered before anesthesia to avoid catabolism. Vitamin supplementation and various coenzyme treatments, such as idebenone, coenzyme Q10, L-carnitine, arginine, and lipoic acid [21, 27, 28], canimprove energy and glycolipid metabolism while applying free radical scavenging drugs, low-dose hormones, and cyclosporine A canimprove autophagy. Patients with encephalopathy should be treatedwith folic acid supplements [29], and arginine also needs to be supplemented when insufficient. Intravenous injection of arginine during stroke-like attacks can improve patients’ headaches, nausea and vomiting, consciousness, and audio-visual impairment [30]. Symptomatic treatment was also important: patients with ptosis, strabismus, and cataracts can obtain long-term results from surgery [31].Gene (rAAV2-ND4)therapy showed complementary neuroprotective effects in LHON m.1178G > A patients [19, 32]. Table 4 shows a summary of the therapeutic improvements and symptoms.

**Table 4:** Therapeutic effects improvement/symptoms

	TX	CPEO (*N* %)	MELAS (*N* %)	KSS (*N* %)	MIDD (*N* %)	LHON (*N* %)	Total (*N *%)
Otolaryngology							
Hearing loss	HA/CI	1/6(16.7)	1/5(20)	1/4(25)	1/4(25)	0/0(0)	4/19(21.1)
Tinnitus	HA/CI	0/1(0)	1/2(50)	0/2(0)	1/1(100)	0/0(0)	2/6(33.3)
Auditory agnosia	RX	0/0(0)	1/1(100)	0/0(0)	0/0(0)	0/0(0)	1/1(100)
Dysphagia	MILL’S	1/5(20)	0/0(0)	0/1(0)	0/0(0)	0/0(0)	1/6(16.7)
Facial weakness	N	0/4(0)	0/0(0)	0/0(0)	0/0(0)	0/0(0)	0/(0)
Ophthalmology							
Ptosis	CFS	8/15(53.3)	0/1(0)	2/5(40)	0/0(0)	0/0(0)	10/21(47.6)
Extraocular muscle paralysis	EOMS	6/12(50)	0/1(0)	1/5(20)	0/0(0)	0/0(0)	7/18(38.9)
Retinitis pigmentosa	IDBN/GT	0/0(0)	0/1(0)	1/4(25)	0/0(0)	1/3(33.3)	2/8(25)
Optic atrophy	IDBN/GT	0/0(0)	0/2(0)	0/3(0)	0/0(0)	2/3(66.7)	2/8(25)
Cataract	PHACO + IOL	3/3(100)	1/3(33.3)	0/0(0)	0/0(0)	0/0(0)	4/6(66.7)
Eye pain	RX	0/0(0)	3/3(100)	0/0(0)	0/0(0)	0/0(0)	3/3(100)
Hemianopsia	N	0/0(0)	0/3(0)	0/0(0)	0/0(0)	0/0(0)	0/3(0)
Glaucoma	RX	1/1(100)	1/2(50)	0/0(0)	0/0(0)	0/0(0)	2/3(66.7)
Diplopia	RX	1/2(50)	0/0(0)	0/0(0)	0/0(0)	0/0(0)	1/2(50)
Neurology							
Exercise intolerance	RX	1/10(10)	1/2(50)	1/4(25)	0/0(0)	0/0(0)	3/16(18.8)
Stroke-like episode	RX	0/0(0)	5/5(100)	0/0(0)	0/0(0)	0/0(0)	5/5(100)
Mental retardation	N	0/0(0)	0/1(0)	0/2(0)	0/2(0)	0/0(0)	0/5(0)
Headache	RX	0/0(0)	3/3(100)	0/0(0)	0/0(0)	0/0(0)	3/3(100)
Palpitation	RX	0/3(0)	0/3(0)	1/3(33.3)	0/0(0)	0/0(0)	1/9(11.1)
Muscular atrophy	N	0/4(0)	0/1(0)	0/2(0)	0/0(0)	0/0(0)	0/7(0)
Myoclonic seizures	RX	0/0(0)	1/4(25)	0/0(0)	0/0(0)	0/0(0)	1/4(25)
Abnormal mental behavior	N	0/0(0)	0/2(0)	0/2(0)	0/0(0)	0/0(0)	0/4(0)
Dizziness	RX	0/0(0)	0/0(0)	0/0(0)	2/3(66.7)	0/0(0)	2/3(66.7)
Limb numbness	RX	0/0(0)	0/0(0)	0/0(0)	2/3(66.7)	0/0(0)	2/3(66.7)
Aphasia	RX	0/0(0)	1/2(50)	0/0(0)	0/0(0)	0/0(0)	1/2(50)
Insomnia	RX	0/0(0)	0/0(0)	1/1(100)	0/0(0)	0/0(0)	1/1(100)
None-Neuromuscular system							
Diabetes	RX	0/0(0)	0/0(0)	0/0(0)	4/4(100)	0/0(0)	4/4(100)
Acute fever	RX	0/0(0)	3/3(100)	1/1(100)	0/0(0)	0/0(0)	4/4(100)
Hypermicrosoma	N	0/2(0)	0/1(0)	0/1(0)	0/0(0)	0/0(0)	0/4(0)
Low BMI	N	0/2(0)	0/1(0)	0/1(0)	0/0(0)	0/0(0)	0/4(0)
Vomit	RX	0/0(0)	2/2(100)	1/1(100)	0/0(0)	0/0(0)	3/3(100)
Diarrhea or constipation	RX	1/1(100)	1/1(100)	0/0(0)	0/0(0)	0/0(0)	2/2(100)
Depression	RX	2/2(100)	0/0(0)	0/0(0)	0/0(0)	0/0(0)	2/2(100)
Kidney damage	RX	0/0(0)	0/0(0)	0/0(0)	1/1(100)	0/0(0)	1/1(100)
Hypogonadism	N	0/1(0)	0/0(0)	0/0(0)	0/0(0)	0/0(0)	0/1(0)

*TX* treatment, *CPEO* chronic progressive external ophthalmoplegia, *MELAS* mitochondrialencephalomyopathy lactic acidosis stroke-like episodes, *KSS* Kearns-Sayre syndrome, *MIDD* maternally inherited diabetes and deafness, *LHON* Leber hereditary optic neuropathy, *HA* hearing aid, *CI* cochlear implantation, *RX* receptor X(coenzyme Q10/ L-carnitine/arginine/lipoic acid), *MILL’S* microlaryngoscopic laser surgery, *N* none, correctin of ptosis andcanthoplasty, *CFS* conjoint fascial sheath, *EOMS* extraocular muscle surgery, *IDBN* Idebenone, *GT* gene therapy, *PHACO* phacoemulsification, *IOL* intraocular lens

## Discussion

### ENT features

The onset of bilateral SNHL with multiple system damage in adolescence can be indicative of MEM. In addition to symmetric progressive SNHL, hearing impairment can also manifest as sudden deafness, acute tinnitus, and auditory agnosia, most of which occur before stroke-like attacks. MEM may be an important cause of idiopathic hearing loss, especially in patients with a maternal family history; The onset age of ENT disorders is later than that of ophthalmology (10–30 years)and neurology (2–31 years) [4, 13, 19]. Facial muscle weakness and dysphagia gradually increasing middle-aged patients (43–35 years) as slow-onset delayed subjective features. Excluding the common diseases in otolaryngology, electromyography and muscle biopsy should be performed.

### Audiology characteristics

The subjective and objective audiological tests conducted in our study is played different sensitivities in the setting of MEM. DPOAE and ABR were more sensitive and reliable than PTA, while AI and CM were not specific. Among the 23 patients studied by Kullar et al. [33], two out of eight patients presented with normal PTA but did not generate OAE, indicating that OAE testing can detect hearing loss earlier than PTA. Objective ABR changes were slightly comparable to DPOAE and less valuable in the setting of LHON. We found that DPOAE was not elicited in 2 of the 13 patients with normal hearing, and DPOAE was not elicited in any patients with moderate or above hearing loss, which is consistent with other reports that involve OAE abnormality as PTA loss  > 40 dB [9]. Among 13 patients with normal PTA, 10 patients with ABR could be elicited, two patients had a threshold shift up, and a single case of wave III latency was prolonged. There have been reports that OAE in MEM with SNHL was normal, but ABR was absent even after neurological symptoms and hearing loss occurred [34]. As for subjective testing, SDS and PTA results matched, and the SDS and PTA decreased disproportionately in only one case. To date, three patients with acute auditory agnosia have been reported with the onset symptoms of MELAS, which requires an audiological evaluation and cranial MRI scan to distinguish it from auditory neuropathy spectrum disorder (ANSD) [35].

### Correlation between SNHL incidence rate and gene penetrance

In our study, 32 patients with MEM were recruited from inpatients, had a neurologically impaired penetrance rate of 56.4–54.4% aural-visual and muscle impairments, and 15.6% had stroke-like attacks. Hearing dysfunction varied with subtypes as follows: CPEO: 53.3%; MELAS: 60%; KSS: 80%; MIDD: 100%; and LHON: 0%. The penetrance of each subtype was different because mutation soccur indifferent genes of mtDNA. According to previous reports, the mutations in the COI/Trna^Ser (UCN)^genes such as mtDNA A7445G, 7472insC, G7444A, and T7511C are almost completely penetrance mutations, and the penetrance of deafness is almost 100%.Withother mutations, such as mtDNA A1555G, C1494T, and other 12SrRNA mutations, the hearing loss depends on nuclear genes and environmental factors; and the penetrance of deafness is 37–87%. In addition to the above mutations that cause non-syndromic deafness, mtDNA mutations encoding mitochondrial proteins often cause aural syndromes, such as MIDD, MELAS, KSS, CPEO, and myoclonus epilepsy with ragged red fibers. Almost all patients with MIDD have hearing loss, and the incidence of hearing loss caused by mtDNA mutations in mitochondrial encephalomyopathy syndrome SNHL is 42–74% [36]. Xia et al. [37] reported that MELAS, CPEO, and other clinically common MEMs have a total incidence of hearing loss of 73.9%; MELAS and KSS are accompanied by 100% hearing loss, and CPEO has the lowest incidence of hearing loss (33.3%). The hearing loss reported by Finsterer et al. [38] in patients with LHON is not common. This study and previous reports have shown that the penetrance rate of aural-visualimpairments (56.2–56.4%) isrelated to the penetrance rate of muscle damage (56.2%), and there is no significant correlation with the age, sex, and progress of the patient. This may be because the level of mitochondrial mutations in cochlear hair cells and vascular lines is consistent with the mtDNA mutation in muscle tissue cells. The level of mitochondrial mutations in muscle tissue cells can reflect cochlear mitochondrial function [38, 39]. The diversity of clinical phenotypes and degrees is associated with mtDNA heterozygosity, and further study of the hearing loss characteristics of MEM would be of value for early detection and monitoring of the disease [40].

### The possibility of lesionsin aural impairment

The audiologist in our study suggested that possible cochlear orretro cochlear lesions are the reason for the observed aural impairments. ABR manifested as 80 dB nHL I, III, or V wave latency extended, ABR thresholds were increased, the range was 40–110 dB HL, and discernible ABR waves could not be detected in severe and profound hearing loss. It has been reported that ABR can display a peak delay between I and III in one ear, and no waveform can be induced in the other ear while OAE, ASR, and the cochlear olive reflexes arenormal, allowing us to conclude that lesions of hair cells and cochlear olive bundles may be excluded as a cause of the observed aural impairment [41]. Most MEM has DPOAE abnormalities with or without ABR abnormalities, suggesting that hearing loss lesions are mainly located in the cochlea. While the lesions were mainly located in the cochlea, they could have been accompanied by pathological changes in the central auditory pathway. However, Maryam et al. [42] found that the acoustic reflection and ABR of two mild SNHL cases were not elicited. Further, two of three MELAS patients had lesions behind the cochlea, with the presumption that the lesions were located in the cochlea and retrocochlear. Lesions of the auditory pathways and auditory cortex often have positive results on cranial MRIs when the central nervous system is involved. Therefore, imaging can help further elucidate the location of the hearing loss [43].

### Pathological mechanisms of SNHL in MEM

Since the audio-visual nerves lack lysosomes and autophagic activity, it is challenging to eliminate mutant mtDNA; thus, the audio-visual impairment increases. The outer hair cells of the cochlea have a high ATP requirement but cannot self-replicate. In particular, cells located at the bottom of the cochlea have the most active metabolism and are extremely sensitive to hypoxia. Hearing impairment in MEM could emerge from end-organ dysfunction due to deficient energy release within the stria vascularis or hair cells, which are metabolically active structures. Hair cells depend on the normal intracochlear potential provided by the stria vascularis, which itself has a high metabolic activity and abundant Na^+^/K^+^-ATP pump. It is assumed that dysfunction of mitochondrial oxidative phosphorylation reduces the level of ATP, thereby progressively imbalances the outer hair cells and stria vascular ions, resulting in cell damage and death. Sudden deafness after a stroke-like attack of MELAS is presumed to be an acute metabolic disorder of the stria vasculature, causing irreversible apoptosis of hair cells. Slowly progressing hearing loss may be due to chronic damage to stria vasculature and hair cells. Auditory conduction pathways and auditory cortical abnormalities can be combined with cranial MRI imaging changes or pathological confirmation of the nervous tissue to clarify the situation. The possible pathological mechanism is nerve demyelination, resulting in unsynchronized nerve cell membrane potential conduction, which may cause some enzyme defects in brain tissue, such as reduction, loss of nerve cells, astrocytic hyperplasia, and microvascular increase [44].

MEM-related facial weakness and dysphagia are often accompanied by ptosis and exercise intolerance for many years, most of which occur in the CPEO and KSS subtypes due to mtDNA fragment deletion [45]. Heighton et al. [46] reported that 90% of CPEO and 40.7% of KSS were accompanied by facial weakness, medulla oblongata, and muscle weakness of the extremities. Facial weakness occurs unilaterally and is the only symptom with a low incidence (3.8%), mostly appearing bilaterally. Medullary palsy manifests as symptoms such as dysphagia and choking when drinking water. Hedermann et al. [12] reported that 56.3% of the drinking cold water tests in 16 CPEOs were abnormal, and the symptoms of dysphagia gradually progressed in patients over 45 years of age. EMG results showed myogenic and/or neurogenic damage that can be accompanied by damage to the skeletal and cardiac muscle groups. When facial weakness and dysphagia occur, in addition to extensive muscle biopsy and gene test, the ECG should be monitored every year to detect early cardiac failure, especially for MELAS and KSS [15, 47].

Monitoring patients’ otolaryngology can have value in detecting the progress of MEM. In the early stages of MEM, the subjective hearing threshold could be normal, but OAE and ABR have been shown to presymptomatically detect abnormalities. Therefore, OAE and ABR monitoring of MEM can be used for early diagnosis/monitoring of pathological progression, which is more valuable than the subjective hearing test. For example, almost 100% of MELAS patients could have a stroke and be in critical condition, but there were no clinical signs before the stroke-like attack. However, there could be OAE and ABR abnormalities. DPOAE, a portable, objective examination widely carried out in clinics, is easier to perform than PTA and ABR. Early detection and early intervention through detection can prevent stroke and reduce morbidity and mortality. ABR abnormalities in the gastrointestinal type of MEM are reportedly related to central injury, so that ABR can be used as indirect evidence of invasion of the gastrointestinal system by MEM [48]. Further study of the correlation between ABR and pathological progress, followed by a judgment of the prognosis through audiological monitoring, could thereby improve the quality of patients’ lives. In addition, facialmuscle weakness and difficulty swallowing suggest muscle damage; muscle biopsy could be performed for classification and symptomatic treatment. However, muscle biopsy alone cannot be used to diagnose mitochondrial disease because many nuclear gene mutations, such as in CPEO, do not exhibit morphological changes in skeletal muscle [39, 46].

### Treatments and results

Currently, a combination of drugs and surgery is being used to treat these syndromes. The main goals are to prolong independence, prevent complications and improve quality of life. Coenzyme Q10 was used to treat MELAS patients for a period of time, and it was found that it can promote insulin secretion, delay hearing loss, improve myopathy symptoms, congestive heart failure, and other complications [49]. Carlot [28] presented a potential therapeutic effect of idebenone treatment for  > 12 months in 53 chronic LHON patients. Other cofactors, including carnitine and vitamins B, C, and K, can also improve ATP synthesis in some mitochondrial diseases [50]. Other drugs harmful to mitochondrial function should be used with caution, including antibiotics such as tetracycline and chloramphenicol and antiepileptic drugs such as phenytoin, sodium valproate, and antiretroviral drugs. Hearing aids are preferred for patients with hearing loss. If the neural pathways and functions are complete, hearing aids are not ideal for hearing, and cochlear implantation can be considered. The first reported case of a patient with mitochondrial disease who received a cochlear implant had KSS. Three patients wore hearing aids, and one case of KSS cochlear implantation was performed in our ENT clinic, and the postoperative results were satisfactory. Mitochondrial disease is not complicated by inner ear deformity and auditory nerve dysplasia, and it causes mostly post-lingual deafness in adults [51]. The number of cochlear spiral ganglion cells in patients with the common mtDNA A1555G mutation remains significant, and the effect of cochlear implantation mainly depends on the residual number of the spiral ganglion and the integrity of the auditory nerve [52]. Doerfer et al. [53] reported the postoperative effects of 25patients with mitochondrial deafness implanted with cochlear implants, all of which had severe SNHL. Although the age of hearing loss and the age of surgery differed, 58% of the patients could talk on the phone after the operation, and the remaining patients had good open sound field speech discrimination scores with no postoperative complications. Okazaki et al. [54] reported that patients with weak facial muscles could undergo fascia or muscle transplantation to improve symptoms. In addition, Mohannak et al. [23] reported that patients with dysphagia could undergo partial pharyngectomy to avoid social disorders, malnutrition, and aspiration pneumonia. Future drug treatments include activation of mitochondrial biosynthesis, mitochondrial autophagy and kinetic regulation, and biochemical defect bypasses. Future targeted therapy could include removal of toxic substances, deoxynucleoside, and deoxynucleotide treatment [55]; gene therapy [32]; follicular plasma replacement therapy [33]; alteration of mtDNA heterogeneity; and stable mutation of mitochondrial mtDNA [56]. The use of blood stem cell transplantation to treat mitochondrial neurogastrointestinalencephalomyopathy has gradually started to emerge clinically [57], and other treatments are still in the preclinical stage.

In conclusion, correlation of clinical, electroneurographical data, structural imaging data, and muscle and gene testing must be relied upon by clinicians to assess MEM patients. Although it is still unclear how mtDNA mutations lead to clinically significant impaired penetrance characteristics and tissue specificity, the severe heterogeneity of clinical phenotypes prompts us, the core symptoms of MELAS syndrome are stroke-like attacks. The main symptoms of CPEO and KSS are eyelid ptosis and extraocular muscle paralysis; it is not uncommon to report atypical symptoms as the first manifestation. In our study, hearing impairment can occurred accompany stroke-like attacks, even when asymptomatic, as detected by OAE and ABR. Dysphagia and facial weakness rarely occurred as the initial presentation, and they were often accompanied by ptosis and exercise intolerance in CPEO/KSS. The disease is relentlessly progressive, with ablepsia, aphasia, dysphagia, tetraparesis, and heart failure potentially occurring with time. There is no specific treatment for MEM. Idebenone, an antioxidant, is the only FDA-approved drug for LHON but has only a modest effect on survival. The multiplicity and progressiveness of the disabilities in MEM highlight the need for a coordinated MDT to manage patient symptoms and nutrition, communication, and physical and occupational therapy. Thus, MDT is ideally suited for both clinical bedside assessment and future multicenter clinical trials in MIDs. In addition, comprehensive discussions about protein expression and pathogenic gene regulationmechanisms are worth exploring to aid in future studies of MEM.

## Data Availability

The data used to support the findings of this study are available from the corresponding author upon reasonable request.

## Abbreviations

MEM: Mitochondrial encephalomyopathy
ENT: Ear, nose, and throat
MIDs: Mitochondrial disorders
MDT: Multidisciplinary team
PTA: Puretone average testing
SNHL: Sensorineural hearing loss
ABR: Auditory brainstem response
AI: Acoustic immittance
ASR: Acoustic stapedius reflex
SPL: Sound pressure level
DPOAE: Distortion product otoacoustic emissions
CM: Cochlear micropotential
SDS: Speech discriminationscore
MST-Ms: Mandarin speech audiometry testing materials
ENoG: Electroneurography
EMG: Limb electromyography
ECG: Electrocardiogram
CPEO: Chronic progressive external ophthalmoplegia
MELAS: Mitochondrialencephalomyopathy lactic acidosis and stroke-like episodes
KSS: Kearns-Sayre syndrome
MIDD: Maternally inherited diabetes and deafness
LHON: Leber hereditary optic neuropathy
N: Normal
Ab: Abnormal
T: Tympanogram
VEP: Visual evoked potential
SDH: Succinate dehydrogenase
COX: Cytochrome oxidase
